# Condensed Fuzheng extract increases immune function in mice with cyclophosphamide‐induced immunosuppression

**DOI:** 10.1002/fsn3.2982

**Published:** 2022-07-18

**Authors:** Ji‐Da Wang, Li Wang, Shuang Yu, Yu‐Tong Jin, Yi‐Yang Wang, Run‐Dong Chai, Ze‐Yu Zhao, Yu‐Hong Bian, Shu‐Wu Zhao

**Affiliations:** ^1^ School of Intergrative Medicine Tianjin University of Traditional Chinese Medicine Tianjin China; ^2^ Pharmaceutical Department Tianjin Second People's Hospital Tianjin China; ^3^ School of Intergrative Medicine Tianjin University Tianjin China

**Keywords:** cellular immunity, condensed Fuzheng extract, cyclophosphamide, immune‐enhancement

## Abstract

Our general purpose was to examine the effect of condensed Fuzheng extract (CFE) on the alleviation of immunosuppression. A mouse model of immunosuppression was established by intraperitoneal injection of CTX. A healthy control group received no CTX and no CFE; different intragastric doses of CFE were administered to three groups of mice for 28 days (4500, 2250, or 1125 mg/kg/day); a negative control received CTX alone, and a positive control received CTX and levamisole hydrochloride. We evaluated the effects of CFE on the immune system organs, cells, and molecules by comparing the different groups. CFE significantly improved immune system organs (spleen and thymus indices and histology), stimulated immune cell activities (number of white blood cells and lymphocytes, phagocytosis of mononuclear phagocytes, proliferation of splenic lymphocytes, antibody formation, and NK cell activity), and increased the levels of immunoglobulins (IgA, IgG, and IgM) and cytokines (IL‐2 and IFN‐γ). Thus CFE effectively alleviated CTX‐mediated immunosuppression and oxidative stress and enhanced the immunological functions of mice.

## Graphical abstract

Cyclophosphamide is widely used but has many toxic and side effects, so we evaluated the immune organs, immune cells, and immune molecules to explore whether CFE has immune improvement effect.

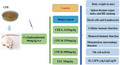

## 1 INTRODUCTION

The human immune system functions in surveillance, defense, and physiological regulation, and these functions are mediated at the levels of organs (e.g., spleen, thymus, and bone marrow), cells (e.g., lymphocytes, mononuclear phagocytes, and neutrophils), and molecules (e.g., antibodies, lysozymes, complements, and immunoglobulins) (Sattler, 2017). The many layers of defense in the immune system provide important protection from external attacks. The immune system functions in innate immunity (nonspecific immunity) and adaptive immunity (specific immunity) and in adaptive immunity, which is further divided into humoral immunity and cellular immunity (Fuentes et al., 2017). Immunosuppression makes the body more vulnerable to diseases (Brodin & Davis, 2017) and can be caused by aging, disease, or medical treatments (Gombart et al., 2020). Therefore, it is crucially important to identify drugs or other treatments that can effectively and safely enhance the immune system (Jabs, 2018).

Cyclophosphamide (CTX) is a broad‐spectrum anticancer drug that has significant efficacy against leukemia and multiple solid tumors. However, CTX can also lead to bone marrow suppression, immunosuppression, inflammation, and other adverse effects when given to cancer patients (Fernández‐Juárez et al., 2021). In particular, CTX can lead to impaired immune cell function, weight loss, decreased function of the spleen and thymus (Attia et al., 2021), and a decreased ratio of red‐to‐white blood cells (WBCs), all of which contribute to an increased risk of infectious disease (Zsiros et al., 2021). Therefore, a safe and effective treatment that enhances immune function may help protect cancer patients from the adverse effects of CTX.

Traditional Chinese medicines (TCMs) have long been used in China and other Asian countries to treat patients with multiple diseases (Yang et al., 2021). Recent studies showed that TCMs and the classic compound preparations, such as Buzhong Yiqi Decoction (Li et al., 2022), can significantly improve immune function and also provide antitumor and antioxidative effects (Wang et al., 2021). Condensed Fuzheng extract (CFE) is a traditional Chinese compound medicine that consists of sheep placenta, *Astragalus membranaceus*, and *Polygonatum*. CFE is commonly used as a supplement for cancer patients to provide relief from fatigue and leukopenia (caused by radiotherapy and chemotherapy) and to increase immune function. There is also evidence that CFE can alleviate mental health problems, such as stress and anxiety, and is especially effective in providing relief from fatigue and weakness. Recent researches on the pharmacological effects of sheep placenta have demonstrated its medicinal value (De et al., 2011). The applications of sheep placenta have expanded, and it is now used to treat many medical problems, such as adrenocortical insufficiency, backache, impaired immunity, depressive symptoms, baldness, and senescence (Muluye et al., 2016).

Many studies have demonstrated that topical application of sheep placenta provides alimentation to the skin and has antiaging effects. Other studies have reported several biological activities of *A. membranaceus* and *Polygonatum*, the other components of CFE. For example, *A. membranaceus* is widely used in Chinese medicine to treat diseases such as compromised immune function and cancer, and evidence suggests it has significant antioxidant and inflammatory effects (Zhang et al., 2020) and also improves immune function (Shen et al., 2021). *Polygonatum* has antitumor effects, and is widely used in clinical settings because of its anti‐osteoporotic, neuroprotective, immunomodulatory, antidiabetic, antifatigue, and other effects (Cai et al., 2021).

In this study, we established a mouse model of immunodeficiency and then examined the effect of CFE on immune function by examination of immune organs, cells, and molecules.

## 2 MATERIALS AND METHODS

### 2.1 Materials and reagents

CTX was from Tianjin Best Seth Biotechnology Co., Ltd. (Tianjin, China), RPMI‐1640 growth medium was from Beijing Solarbio Science & Technology Co., Ltd. (Beijing, China), concanavalin A (ConA) was from Sigma‐Aldrich (Shang Hai, China), the CCK‐8 Cell Proliferation and Cytotoxicity Assay Kit was from Beijing Solarbio Science & Technology Co., Ltd, and ELISA kits that measured immunoglobulin‐A (IgA), immunoglobulin‐G (IgG), immunoglobulin‐M (IgM), interleukin‐2 (IL‐2), and interferon‐γ (IFN‐γ) were from Multisciences (Lianke) Biotech, Co., Ltd. (Hangzhou, China).

### 2.2 Preparation of condensed Fuzheng extract

CFE was provided by the Mongolian Medicine Hospital of Alxa League in Inner Mongolia. The production batch number was 20,200,615, and the preparation process had a national invention patent (202110881011.5). For preparation, 35 kg of sheep placenta and 35 kg water were added together, and protease fermentation was performed for 24 h. Then, 2 kg of *A. membranaceus* and 2 kg of *Polygonatum sibiricum* were added to 120 kg water and boiled for 4 h. The fermentation broth of the sheep placenta (75 kg) was then added, the mixture was centrifuged at 9000 rpm for 30 min, the supernatant was isolated, and 2 kg brown sugar was added. The solution was boiled and concentrated for 2 h until it became a paste with a concentration of 80%.

### 2.3 Animals

Male mice (19–21 g) that were specific pathogen free (SPF) and specific bacteria free (SBF) were from the Institute of Cancer Research Biotechnology Co., Ltd. (Beijing, China; Ltd SCXK, [Jing] 2019–0010). All mice were maintained at 24.5°C ± 0.5°C with a 12 h light/dark cycle and received food and water ad libitum. All animal experiments were approved by the Experimental Animal Welfare Ethics Committee of Tianjin University of Chinese Medicine (TCM‐LAEC2020046).

### 2.4 Experimental grouping and CFE administration

Mice were randomly divided into six groups, with six mice per group: a healthy control group (CG), model group (MG), CFE high‐dose group (CFE‐H), CFE medium‐dose group (CFE‐M), CFE low‐dose group (CFE‐L), and levamisole hydrochloride group (LYC, positive control). On days 1, 2, and 3, all mice in the MG, CFE‐H, CFE‐M, CFE‐L, and LYC received intraperitoneal injections of 80 mg/kg CTX, and mice in the CG group received intraperitoneal injections of the same amount of saline. On the 4th day, intragastric CFE was administered each day for 28 days to mice in the CFE‐H group (4500 mg/kg), the CFE‐M group (2250 mg/kg), and the CFE‐L group (1125 mg/kg); intragastric LYC (10 mg/kg) was administered on the same schedule to mice in the LYC group, and the same amount of intragastric saline was given to the MG. The CFE‐M doses were based on the clinical dose of 15 g per day used for an adult who weighs 60 kg, and the LYC dose was based on previous studies. After the final treatment, all mice were euthanized using anesthesia.

### 2.5 Amino acid quantification and data analysis

Amino acids were measured by ultra‐high‐performance liquid chromatography (UHPLC) with mass spectrometry. First, 100 mg of ileal contents was mixed with 1 ml acetonitrile/methanol/water (2:2:1, v/v/v) and sonicated for 30 min (4°C), followed by centrifugation (15,000 rpm, 4°C, 20 min). Then 50 μl of plasma and 200 μl of methanol acetonitrile/methanol (1: 1, v/v) were mixed. This mixture was allowed to stand for 60 min before centrifugation (15,000 rpm, 4°C, 20 min). Then, the supernatant was collected and added to the UHPLC (Agilent, Santa Clara, CA, United States) that was coupled with a mass spectrometer (QTRAP 5500 system, Sciex, Redwood, VA, United States). If the relative standard deviations of the quality control (containing the same amount of contents from each sample) for all amino acids were less than 30%, the measurements were considered reproducible and stable (Han et al., 2019).

### 2.6 Determination of thymus and spleen indices and histopathology

After euthanasia, the thymuses and spleens were removed from all mice, rinsed with a normal saline solution, dried with absorbent paper, and then weighed. The index for each organ was calculated from the organ weight (g) and mouse weight (g) on the day of sacrifice as:

Organ Index = (organ weight)/(mouse weight) × 100.

The organs were then prepared for histopathologic examinations by rapid fixation in a 10% buffer solution. Then 5‐μm sections were prepared using a microtome (Leica RM2235) and stained with hematoxylin and eosin (H&E). The histology of organs in the different groups was observed and recorded under a microscope as previously described (H. Liu et al., 2019).

### 2.7 Determination of NK cell activity and routine blood tests

#### 2.7.1 NK cell activity

After euthanasia, the mice were immersed in 75% ethanol for 3 min. Then, their spleens were removed, rinsed with phosphate‐buffered saline (PBS), passed through a 200‐mesh sieve, and centrifuged (1500 rpm, 10 min). To prepare “effector cells,” a suspension of splenic lymphocytes was prepared at 2.5 × 107 cells/ml and was added to RPMI‐1640 culture medium that contained 0.5% bovine serum albumin (BSA). To prepare “target cells,” a suspension of YAC‐1 cells (5 × 105cells/ml) was resuspended in RPMI‐1640 medium that contained 0.5% BSA. Then 100 μl of effector cells and 100 μl target cells (50:1 ratio) were added to 96‐well plates. Target cells (100 μl) and RPMI‐1640 medium containing 0.5% BSA (100 μl) were added to the control well. Target cells (100 μl) with 20% NP‐40 lysate (100 μl) were added to the maximum release hole. Then, the different mixtures were cultured at 37°C with 5% carbon dioxide for 4 h, centrifuged (1500 rpm, 5 min), and 100 μl of the supernatant was extracted from each well and placed into a separate 96‐well plate. Then 100 μl of an LDH matrix solution and 300 μl of a 1 mol/L HCl solution were simultaneously added to each well for 3 min, and absorbance (A) was measured at 450 nm. NK cell activity was then determined as previously described (Sun et al., 2018):

Activity (%) = (A_Reaction hole 1_ − A_Natural release hole_)/(A_Maximum release hole_ − A_Natural release hole_) × 100%.

#### 2.7.2 Routine blood tests

All animals were fasted overnight prior to blood tests, but were allowed to drink water freely. The mice were anesthetized, and blood was collected from the orbital sinus. The complete blood was gathered in an EDTA test tube containing and used immediately for blood analysis. Hematological analysis was conducted using an automatic blood cell analyzer (Mindray® BC‐2800Vet, Guang Zhou, China) as previously described (Z. Huang et al., 2019).

### 2.8 Determination of immune cell function

#### 2.8.1 Proliferation of spleen lymphocytes

The spleens were prepared as described above. After discarding the supernatant, the precipitate was mixed with a 3× volume of erythrocyte lysate, placed on ice for 15 min, and then centrifuged (1500 rpm, 10 min). Then, the supernatant was discarded and RPMI‐1640 medium was added to the precipitate. The suspension was centrifuged again (1500 rpm, 10 min) and the precipitate was resuspended in RPMI 1640 medium with 10% fetal bovine serum. The cells were then cultured with 5% CO2 at 37°C for 24 h and primary spleen cells were then counted after methylene blue staining. After this procedure, the percentage of living cells was 90 to 93%. The splenic primary cells were seeded into 96‐well cell culture plates (1 × 10^5^ cells per well) and ConA was added to achieve a final concentration of 5 μg/ml. After incubation for 24 or 48 h, 10 μl of CCK‐8 was added to each well followed by culturing for 4 h. Then, absorbance was measured at 450 nm and proliferation was determined as previously described (Han et al., 2020).

Proliferation = A_cells induced by ConA_ – A_cells without ConA_.

#### 2.8.2 Delayed hypersensitivity

On day 23, two discs (3.0 × 3.0 cm) were cut from the left and right ears of each mouse, and 10 mg/mL 2,4‐dinitrofluorobenzene (DNFB; Lot: F20090726; China Pharmaceutical Group Shanghai Chemical Reagent Co., Ltd.) was smeared onto the shaved regions. 5 days after establishment of an allergic response, both sides of the right ears were smeared with 10 μl of 10 mg/mL DNFB solution, and the left ears were untreated controls. Then 24 h later, the mice were sacrificed by cervical dislocation, and a round piece of ear (diameter: 8 mm) was collected using a puncher and weighed. The ear swelling was calculated as previously described (Huo et al., 2020):

Ear swelling (%) = (weight of right ear − weight of left ear)/(weight of left ear).

### 2.9 Determination of humoral immunity

The modified glass slide method was used to measure humoral immune function. Sterile sheep blood was collected and washed with sterile PBS three times with centrifugation (2000 rpm, 10 min each). Sheep red blood cells were prepared with normal saline in a 2% (v/v) cell suspension for later use. Each mouse received intraperitoneal inoculations with 0.2 ml sheep red blood cells (SRBCs) at 2% concentration for primary immunization. On the 4th day after immunization, the mice were euthanized, their spleens were collected under aseptic conditions, and a cell suspension was prepared with PBS as above. Finally, the cells were put into PBS.

The prepared surface culture medium was heated until all ingredients were dissolved, and then mixed with the same amount of Hank's solution (pH 7.4) at twice the concentration and added into 0.5 ml tubes. Then, 10% SRBC (50 μl, v/v) and 20 μl of a spleen cell suspension were added into the tube. The solution was quickly mixed and then poured onto the glass slides with a thin layer of agarose. After cooling and solidification, the glass slides were placed horizontally on the bracket and cultured in a CO_2_ incubator for 1.5 h. Then the complement ratio was diluted with SA solution by 1:10. It was then added into the groove of the slides and incubated for about 1.5 h at 37°C for determination of hemolytic plaques as previously described (Oh et al., 2018).

### 2.10 Determination of the function of mononuclear macrophages

On day 24, 0.2 ml of 2% of a saline suspension of chicken erythrocytes was administered to mice by intraperitoneal injection. 4 days later, the mice were sacrificed by cervical vertebral displacement, and each mouse received an intraperitoneal injection with 4 ml of Hank's solution and calf serum. The peritoneal macrophages were fully washed, and then 0.5 ml of peritoneal fluid and 0.5 ml of 1% chicken blood erythrocyte suspension were mixed in test tubes. A 0.5 ml quantity of the mixture was absorbed and added to a 3% agar ring that was dried on a glass slide. The mixture was incubated at 37°C for 20 min, fixed with methanol, and stained with GIMSA solution. Phagocytosis and the phagocytic index were recorded using microscopy as described previously (Rong et al., 2019).

### 2.11 Determination of immunoglobulins and cytokines

Blood from the eyeballs of mice was collected, allowed to sit for 30 min, centrifuged (4°C, 3500 rpm, 10 min), and immunoglobulins and cytokines were measured using ELISA.

## 3 RESULTS

### 3.1 Levels of amino acids in CFE

We used protease during the production of CFE, and the types and concentrations of amino acids are related to immune function. Thus, we first determined the levels of different amino acids in CFE (Table 1). The results showed that the CFE contained many essential and nonessential amino acids.

**TABLE 1:** Concentrations of free amino acids (μmol/g) in CFE

Amino acid	Amount	Amino acid	Amount
Alanine/sarcosine	16.388	Isoleucine2	3.10
Arginine	8.35	Lysine	4.91
Asparagine	7.85	Methionine	0.53
Aspartate	14.53	Ornithine	3.11
Choline	56.16	Phenylalanine	3.38
Citrulline	1.06	Proline	3.29
Glutamate	0.53		
Glycine	32.45		
Histidine	2.06		
Serine	13.48		
Taurine	0.93		
Threonine	12.39		
Tryptophan	1.02		
Tyrosine	15.24		
Valine	7.45		

### 3.2 Effect of CFE on immunosuppressed mice

**FIGURE 1:**
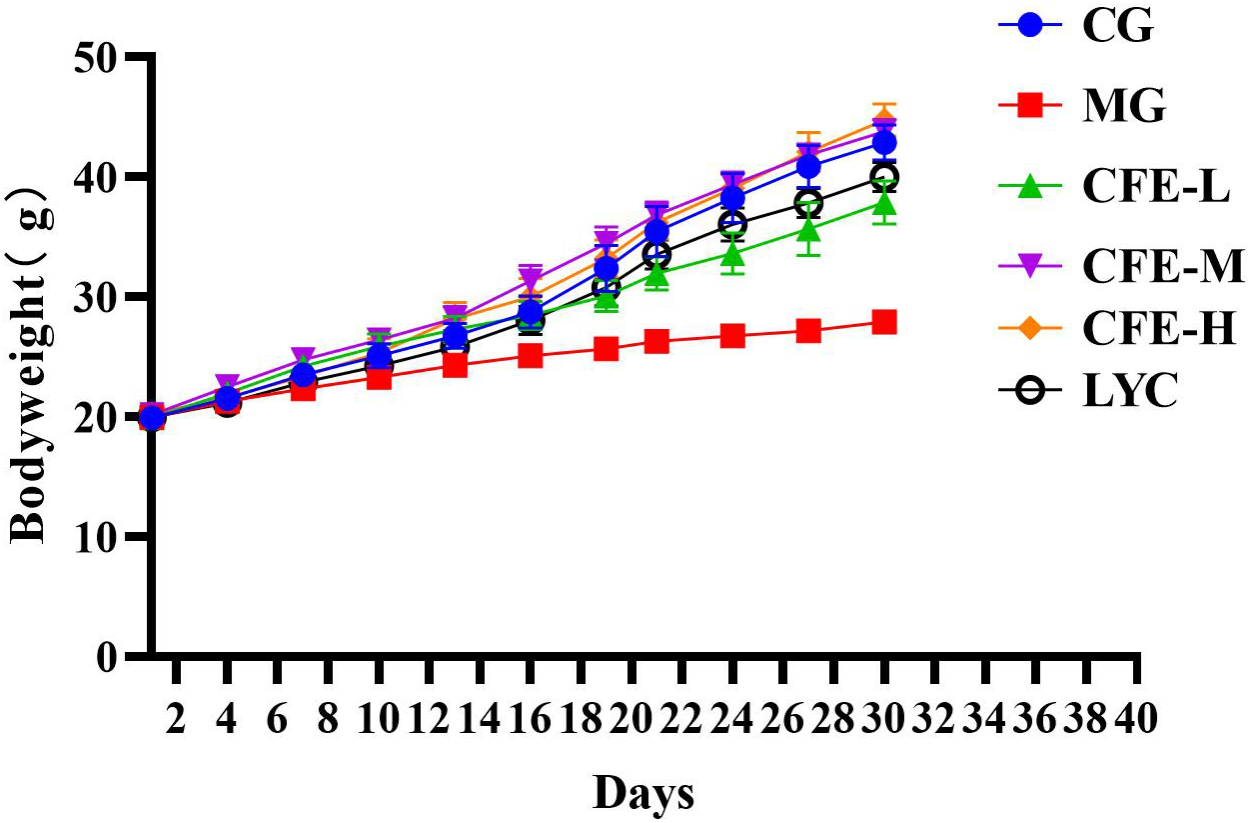
Effect of CFE on the body weight of immunosuppressed mice. The MG mice had a significantly lower weight than the CG mice (*p* < .05), and the CFE‐H, CFE‐M, CFE‐L, and LYC mice had significantly greater weights than the MG mice (all *p* < .05)

We determined the effect of CFE treatment on the body weight of mice that were subjected to CTX‐mediated immunosuppression (Figure 1). On the 30th day, the results show that the weight of MG mice was significantly lower than CG mice (*p* < .05), and that the weights of CFE‐H, CFE‐M, CFE‐L, and LYC mice were significantly greater than that of the MG mice (all p < .05). Thus, CFE effectively reversed the effect of CTX in causing body weight loss in immunosuppressed mice.

**FIGURE 2:**
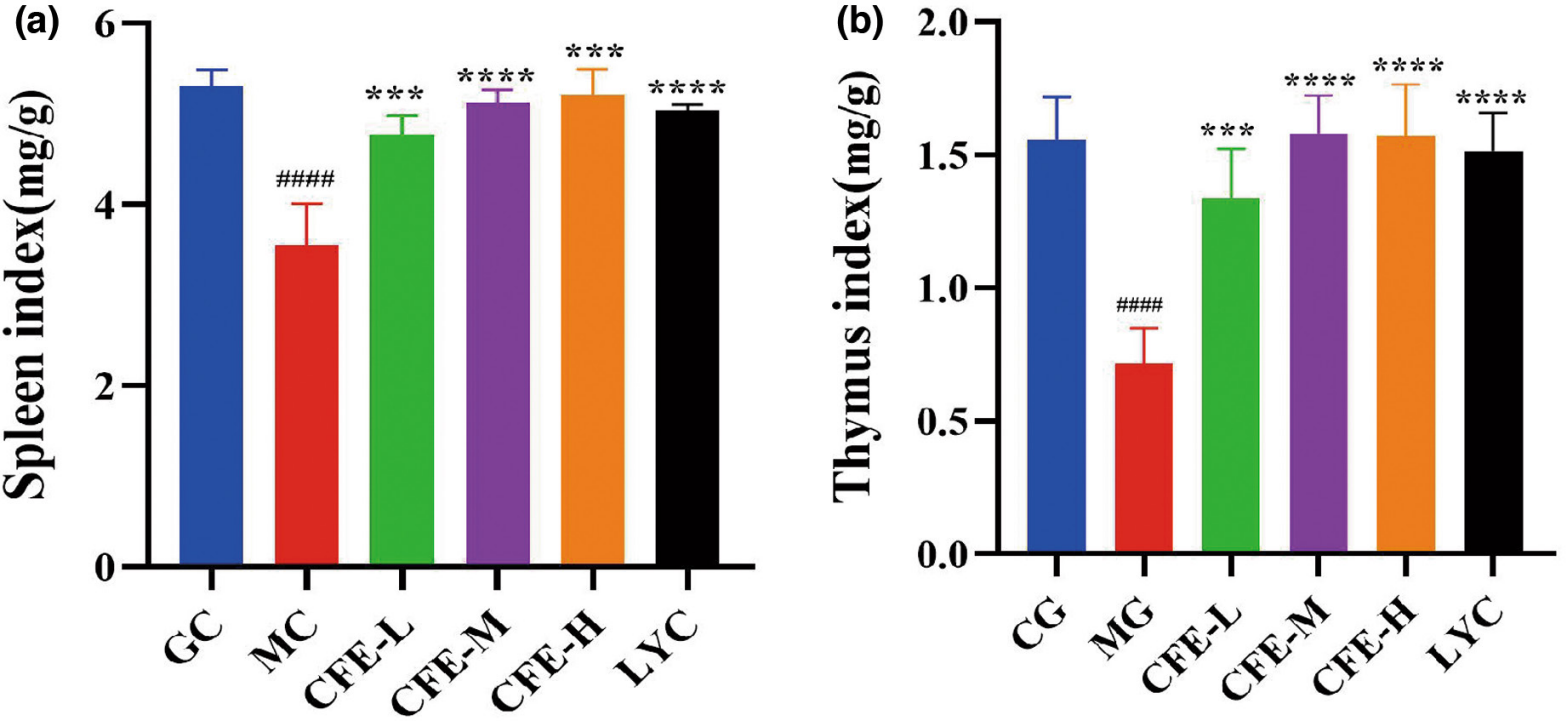
Effect of CFE on the thymus index (a) and spleen index (b) of immunosuppressed mice. For both indices, the values for the MG mice were significantly less than those for the CG, CFE‐H, CFE‐M, CFE‐L, and LYC mice. ***p < .001, ****p < .0001, ^####^ *p* < .0001

We then determined the effect of CFE on the spleen index (Figure 2a) and thymus index (Figure 2b) of immunosuppressed mice. The results show that the spleen index was smaller in the MG mice than in the CG mice (*p* < .0001), and was greater in the CFE‐H, CFE‐M, CFE‐L, and LYC mice than in the MG mice (all *p* < .0001). Similarly, the thymus index was smaller in the MG mice than in the CG mice (*p* < .0001), and was greater in the CFE‐H, CFE‐M, CFE‐L, and LYC mice than in the MG mice (*p* < .0001). These results show that CFE increased the spleen and thymus indices in immunosuppressed mice.

**FIGURE 3:**
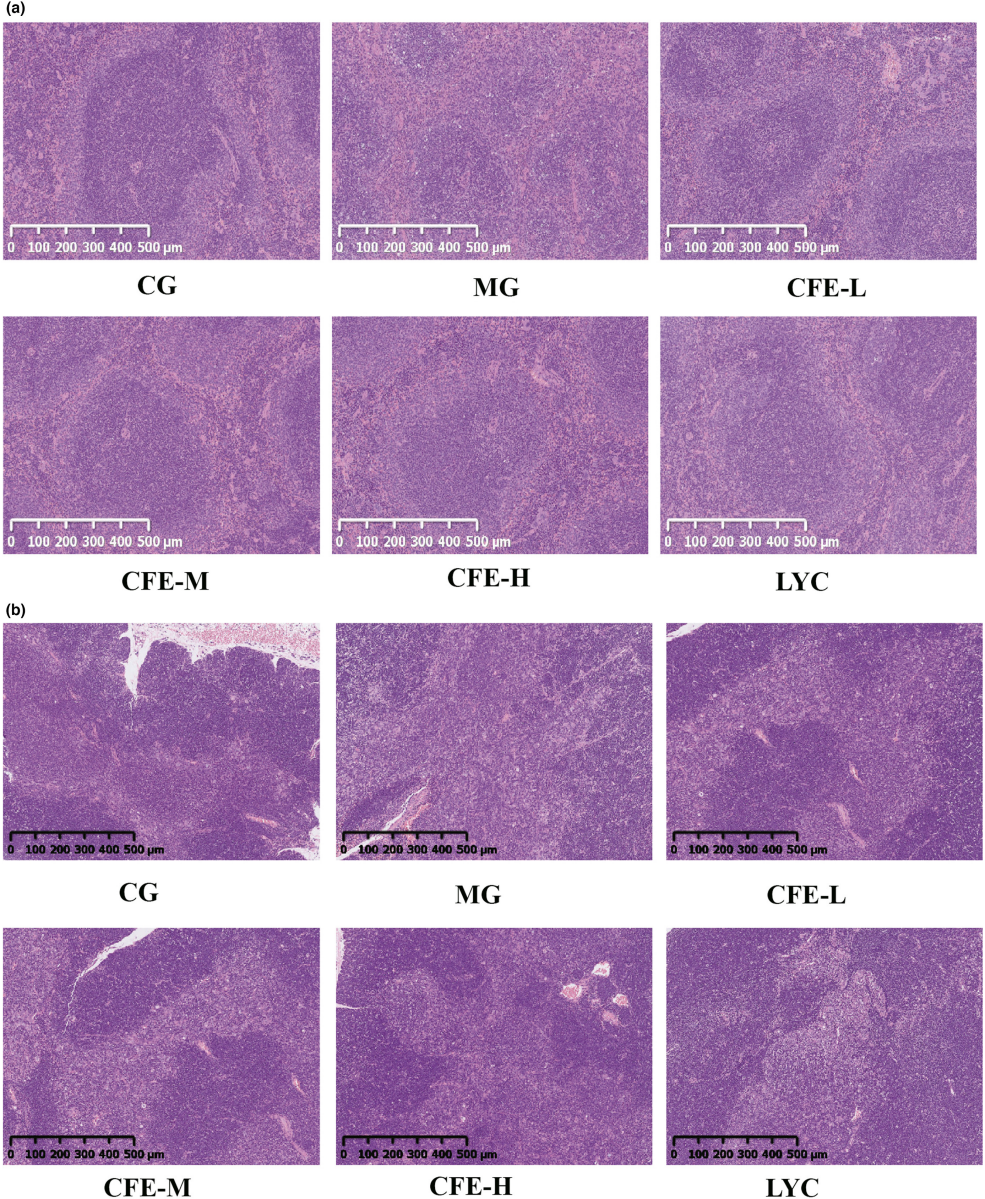
Effect of CFE on spleen histology (a) and thymus histology (b) of immunosuppressed mice

We then examined the effect of CFE on the histology of spleen cells (Figure 3a) and thymus cells (Figure 3b) using H&E staining. Analysis of spleen cells indicated the cells were sparse and irregular in the MG mice, but were tight, well‐arranged, and with clear nuclei in the CG mice. In particular, the red and white pulp of the spleen in the MG mice had unclear boundaries. Specifically, the white pulp was destroyed and the structure was disordered; the trabeculae of the spleen were dense; and there were few lymphocytes. In contrast, the red and white pulp of spleens in the CFE‐H, CFE‐M, CFE‐L, and LYC mice were relatively clear and had many lymphocytes.

Analysis of thymus cells in the CG mice indicated the epithelial tissue and medulla were clearly demarcated, the cortex was dark, the lymphocytes were close together, and the medulla was light. However, the thymus epithelial tissue and medulla in the MG mice were unclear, and atrophy was evident. Compared with MG mice, the thymus epithelium and medulla in the CFE‐H, CFE‐M, CFE‐L, and LYC mice appeared healthier, with dark cortexes and clear boundaries between the epithelium and medulla. Thus, CFE normalized the histology of cells in the spleen and thymus of immunosuppressed mice.

### 3.3 Effect of CFE on immune cell function of immunosuppressed mice

**FIGURE 4:**
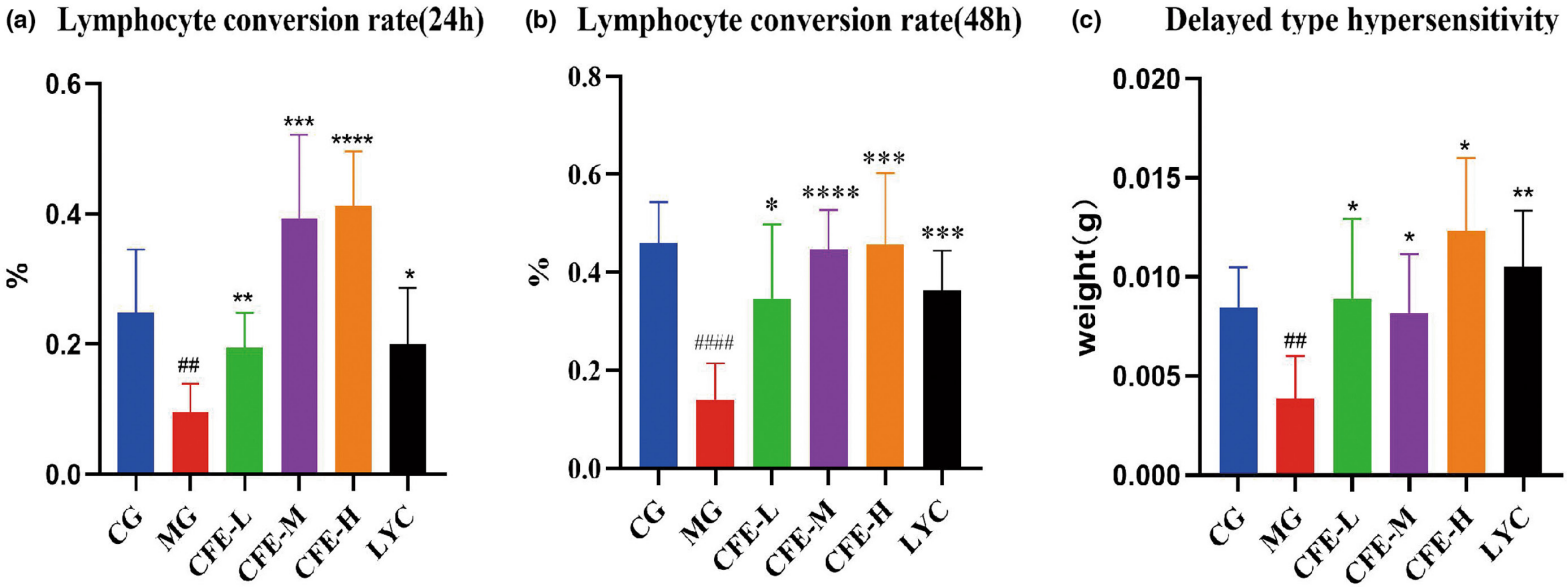
Effect of CFE on the lymphocyte conversion rate at 24 h (a) and 48 h (b) and on delayed hypersensitivity (c) in immunosuppressed mice. For all three variables, the values for the MG mice were significantly less than those for the CG, CFE‐H, CFE‐M, CFE‐L, and LYC mice. *p < .05, **p < .01, ***p < .001, ****p < .0001, ^##^ *p* < .01, ^###^ *p* < .001, ^####^ *p* < .0001

We then examined the effect of CFE on immune cell function. Induction by ConA for 24 h (Figure 4a) and 48 h (Figure 4b) showed that the proliferation of lymphocytes was much lower in the MG mice than in the CG mice (*p* < .01), and that proliferation of lymphocytes was much greater in the CFE‐H, CFE‐M, CFE‐L, and LYC mice than in the MG mice (all *p* < .001).

Analysis of the delayed hypersensitivity response using the ear swelling assay (Figure 4c) indicated this response was much less in the MG mice than in the CG mice (*p* < .01), but that the LYC mice had increased ear swelling and CFE increased ear swelling in a dose‐dependent manner (all *p* < .05).

**FIGURE 5:**
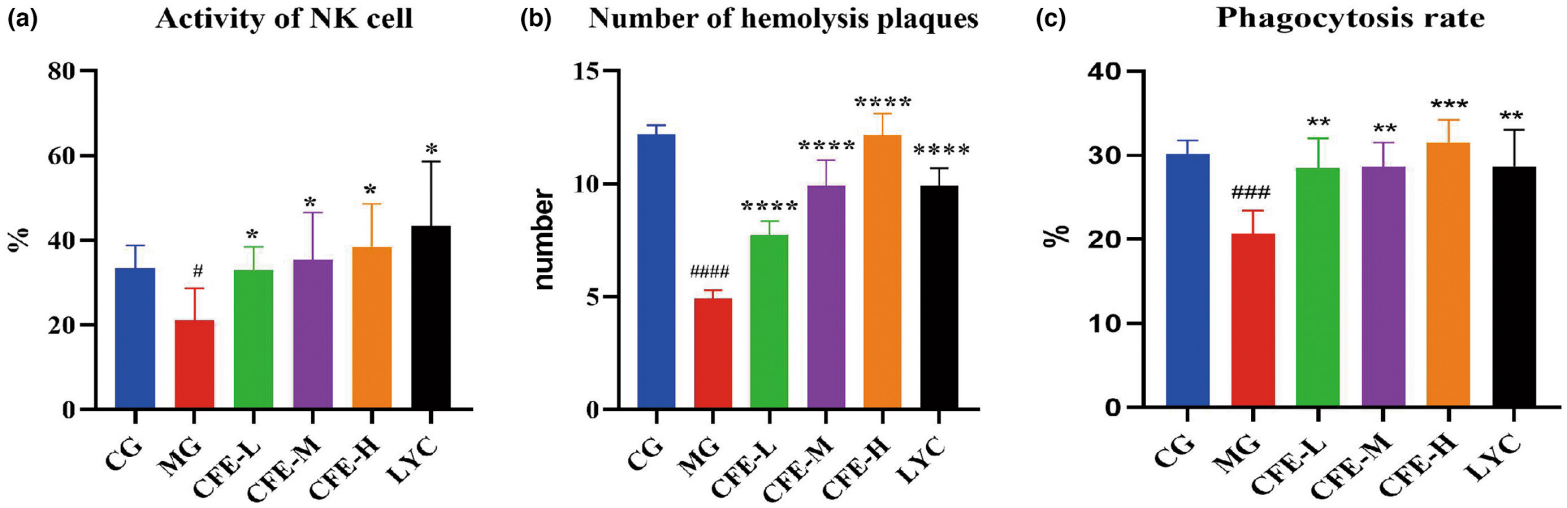
Effect of CFE on the activity of NK cells (a), the number of hemolytic plaques (b), and phagocytosis (c) in immunosuppressed mice. For all three variables, the values for the MG mice, were significantly less than those for the CG, CFE‐H, CFE‐M, CFE‐L, and LYC mice. ^##^ *p* < 0.01, ^###^ *p* < 0.001, ^####^ *p* < 0.0001,*p < 0.05, **p < .01, ***p < .001, ****p < .0001

NK cells with greater activity, a greater number of hemolytic plaques, and a higher rate of phagocytosis reflect greater immune cell function. Relative to the MG mice, the CG mice had significantly greater NK cell activity (*p* < .05, Figure 5a), more hemolytic plaques (*p* < .05, Figure 5b), and a higher rate of phagocytosis (*p* < .05, Figure 5c). Also relative to the MG mice, the CFE‐H, CFE‐M, CFE‐L, and LYC mice had greater NK cell activity, more hemolytic plaques, and a higher rate of phagocytosis (all *p* < .05). Thus, CFE improved the nonspecific immunity and specific immunity in immunosuppressed mice.

**FIGURE 6:**
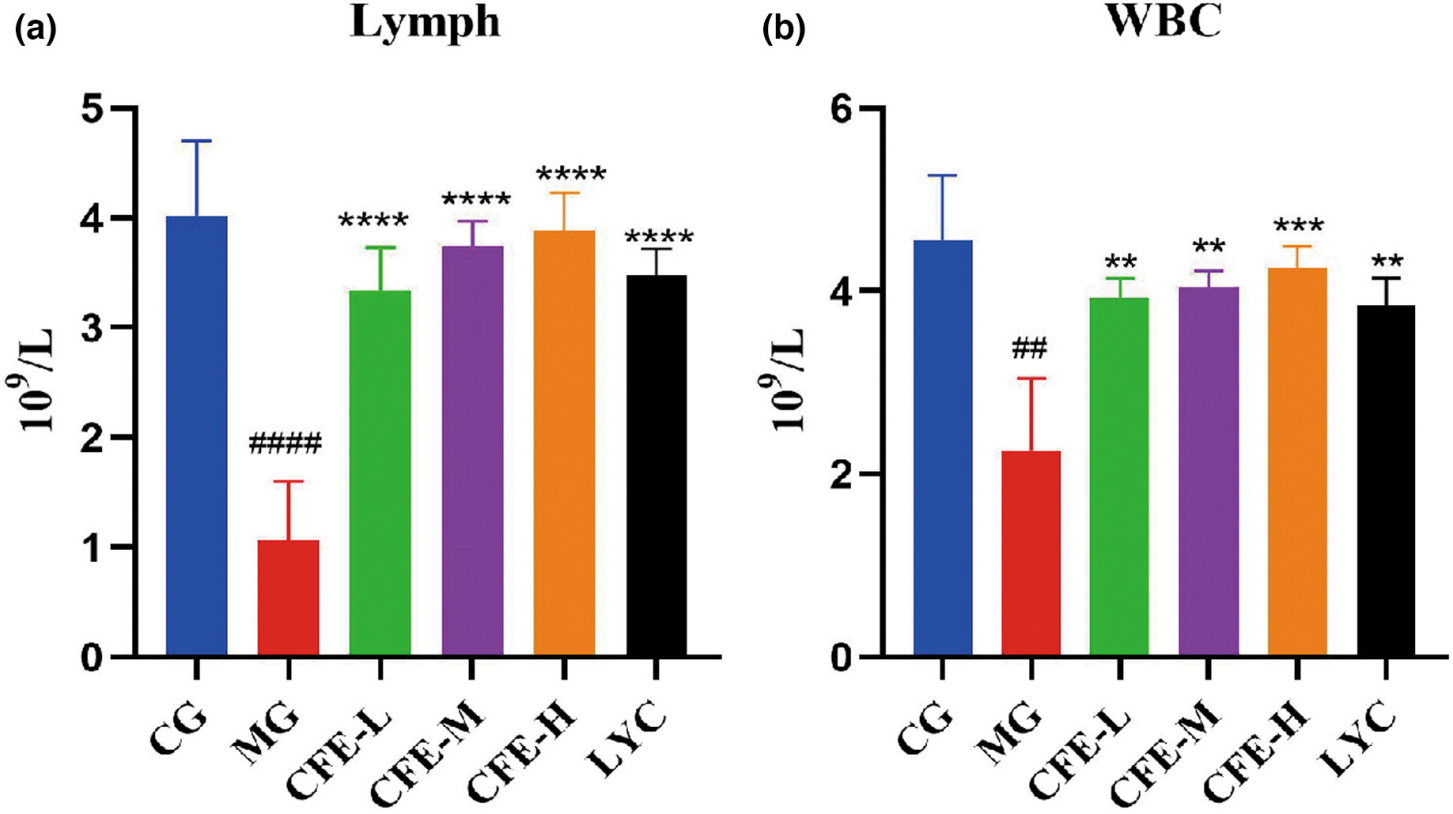
Effect of CFE on the white blood cell count (a) and lymphocyte count (b). For both types of cells, the counts for the MG mice were significantly less than for the CG, CFE‐H, CFE‐M, CFE‐L, and LYC mice. ^##^ *p* < .01, ^###^ *p* < .001, ^####^ *p* < .0001,*p < .05, **p < .01, ***p < .001, ****p < .0001

We measured the effect of CFE on the lymphocyte count (Figure 6a) and total WBC count (Figure 6b) and phagocyt. The results indicated the MG mice had lower levels of WBCs and lymphocytes than the CG mice (both *p* < .05), and the CFE‐H, CFE‐M, CFE‐L, and LYC mice had greater levels of WBCs and lymphocytes than the MG mice (all *p* < .05). Thus, CFE increased the white blood cell and lymphocyte counts in immunosuppressed mice.

### 3.4 Effect of CFE on immune molecules in immunosuppressed mice

**FIGURE 7:**
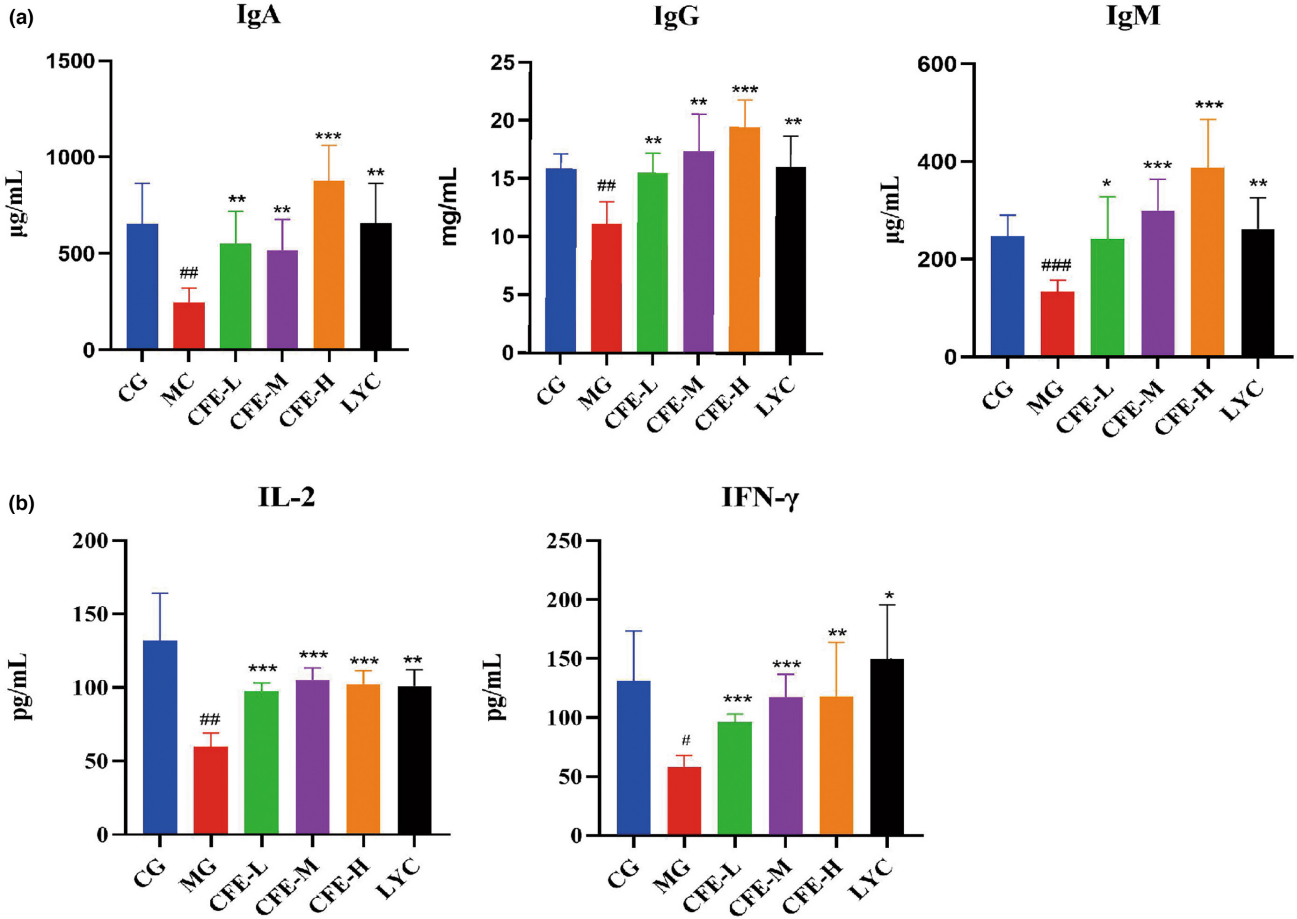
Effect of CFE on the levels of immunoglobulins and cytokines in immunosuppressed mice. For all analytes, the MG mice had significantly lower levels than the CG, CFE‐H, CFE‐M, CFE‐L, and LYC mice. ^##^ *p* < .01, ^###^ *p* < .001, ^####^ *p* < .0001,*p < .05, **p < .01, ***p < .001, ****p < .0001

**FIGURE 8:**
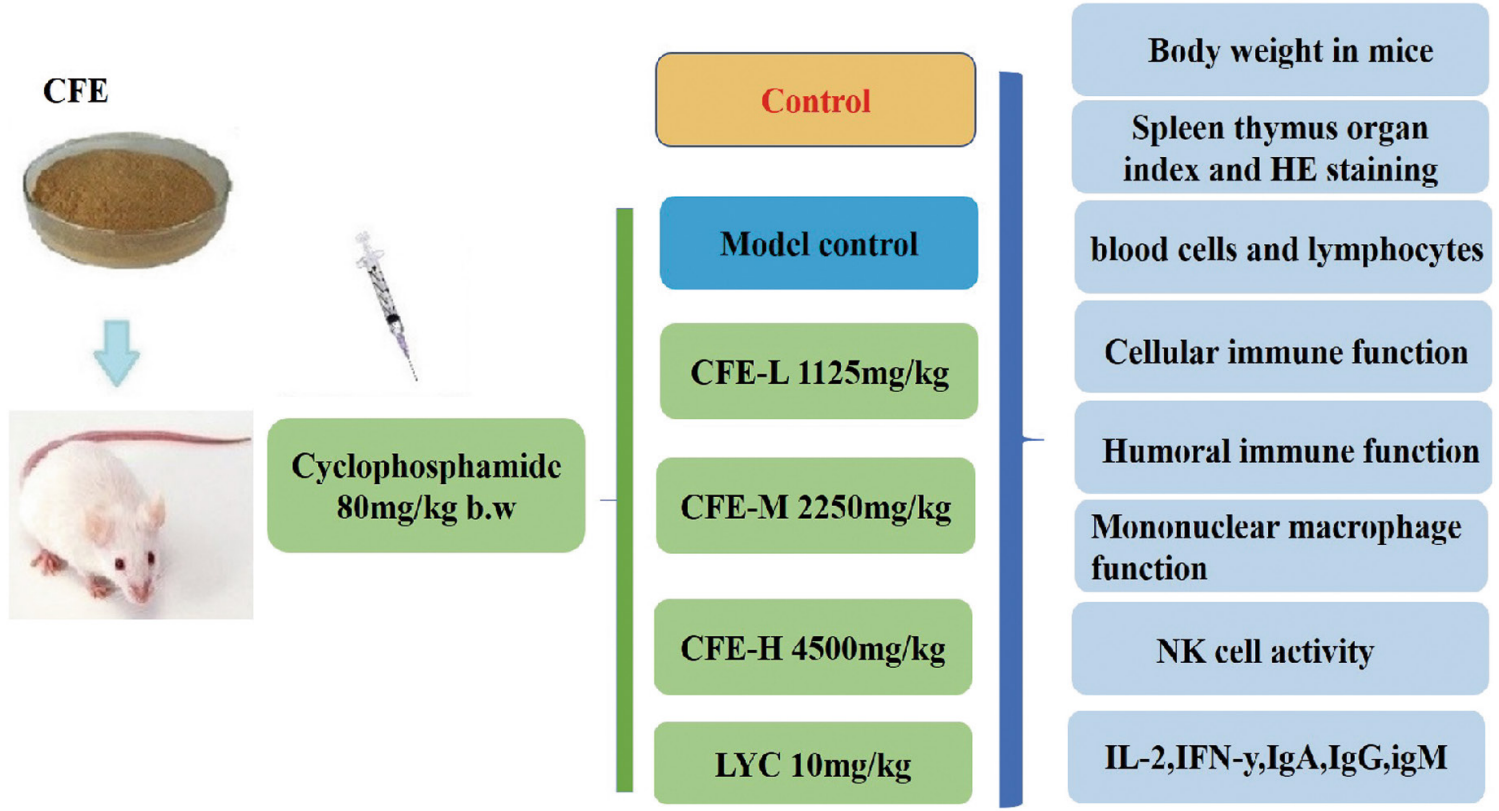
Graphical table of contents

We then used ELISA to measure the levels of three serum immunoglobulins (IgA, IgG, and IgM) and two cytokines (IL‐2 and IFN‐γ) that have important immune functions (Figure 7). The serum levels of all five molecules were lower in the MG mice than in the CG mice (all *p* < .01) and were greater in the CFE‐H, CFE‐M, CFE‐L, and LYC than in the MG mice (all *p* < .05). Thus, CFE increased the levels of immunoglobulin and cytokines in immunosuppressed mice. (Figure 8)

## 4 DISCUSSION

Modern medicine has shown that immune suppression manifests as reductions of innate immunity and acquired immunity. In particular, an immunosuppressed individual has a reduced ability to respond to antigens, changes in the numbers and functions of immune cells, and decreased activities and levels of cytokines. Many studies have identified the causes of immunosuppression (Li et al., 2018), and these include medical treatments, radiation, infection, and others (Ahlmann & Hempel, 2016). CTX is widely used to treat lung cancer, breast cancer, and other cancers. Nevertheless, its use can lead to immunosuppression and an increased risk of infectious diseases. Therefore, a safe and effective immune booster is needed for patients taking CTX (Otterness & Chang, 1976).

TCMs have been used for thousands of years, and certain TCMs are effective in improving the function of the immune system. Modern studies have shown that sheep placenta, *Astragalus* glycosides, and *Rhizoma polygonati* polysaccharides can effectively improve immunity, possibly by regulating the activity of immune cells through the TLR4, MAPK, and other signaling pathways (Niu et al., 2020).

In many cases, the types and concentrations of amino acids are closely related to the immune function (Melano et al., 2021). Amino acids have important effects on physiological functions, in that they can affect metabolism, neurotransmission, and liposome transport. Previous studies showed that many TCMs that are rich in amino acids have immune‐enhancing effects (Kelly & Pearce, 2020). CFE consists of sheep placenta, *A. membranaceus*, and *Polygonatum*, and sheep placenta accounts for 90% of the total. Thus, we used untargeted metabolomics to determine the composition of amino acids in CFE. Our results indicated that CFE has many essential and nonessential amino acids. Many diseases that affect immune function, the nervous system, and the circulatory system are characterized by deficiencies of essential amino acids. There is also evidence that methionine can inhibit the growth of cancer cells (Wanders et al., 2020), that leucine can alleviate depression (Walker et al., 2019), and that threonine can reduce inflammation (Li et al., 2019).

The spleen and thymus have important immune functions, and CTX treatment alters the histology and function of these organs. The thymus and spleen are the sites for immune cell growth and proliferation, and increased weights of these organs indicate increased production of lymphocytes. In turn, a decrease in immune function may be caused by atrophy of these two organs. In our study, the spleen and thymus indices were decreased in mice that had CTX‐mediated immunosuppression. Additionally, our H&E staining showed fewer splenic lymphocytes and disordered structure of the thymus cortex and medulla. Similar results were reported in a previous study of *Fagopyrum esculentum* and longan polysaccharides (Bai, Jia, et al., 2020). However, we found that administration of CFE increased the spleen and thymus indices and also restored the normal histology of these organs.

Lymphocytopenia is a common adverse effect of CTX. WBCs and leukocytes protect against infectious diseases and foreign invaders. Routine diagnostic blood tests are commonly used to determine the distribution, concentrations, and cellular morphological changes of WBCs and lymphocytes. NK cells can specifically recognize tumor cells and foreign cells, and then release cytokines that lead to selective lysis and cell death (H. Wang et al., 2019). We found that the WBC count, lymphocyte count, and NK cell activity were all lower in immunocompromised mice, but that CFE treatment restored these levels to normal.

Lymphocyte proliferation is the most direct indicator of immune status. Lymphocytes have nonspecific mitogen receptors and receptors that recognize antigens, such as ConA. Lymphocytes proliferate and differentiate in vitro or in vivo following mitogen stimulation. In this study, we used ConA to induce lymphocyte proliferation. The results showed inhibition of lymphocytes proliferation in immunosuppressed mice, and that CFE reversed this effect. This result is consistent with a previous study which reported that CFE enhanced the immune function of mice by promoting lymphocyte proliferation (Huang et al., 2020).

Mononuclear macrophages have an important function in nonspecific immunity, and these cells have anti‐infection activities, promote antigen presentation, and initiate the immune responses (Daillère et al., 2016). Macrophages also secrete various cytokines and participate in immune regulation. Thus, we evaluated the function of mononuclear macrophages by measuring the phagocytosis of chicken red blood cells. In agreement with previous studies, we found that CTX reduced the activity of monocyte macrophages, and that CFE significantly reversed this effect (Chen et al., 2021).

Cytokines and immunoglobulins also have important functions in immune responses, and we therefore determined the effect of different treatments on the levels of these molecules. Some evidence suggests that the polysaccharides or peptides in TCMs improve immune function in immunosuppressed mice by activating immune cells and increasing serum cytokine levels. Our results showed that CFE significantly promoted the expression of IL‐2, IFN‐γ, IgA, IgG, and IgM. IL‐2 stimulates the proliferation and differentiation of lymphocytes and increases the cytolytic activity of NK cells and the secretion of IFN‐γ (Y. Liu et al., 2021). IFN‐γ is one of the main immunomodulatory molecules that promote the immune response of macrophages. Immunoglobulin is produced by B lymphocytes following stimulation by pathogenic microorganisms, and it functions in the elimination of these pathogens (Karki et al., 2021).

## 5 CONCLUSION

Our results demonstrated that CFE treatment significantly improved spleen and thymus morphology, histology, and function in mice with CTX‐mediated immunosuppression. At the cellular level, CFE increased the numbers of WBCs and lymphocytes, and phagocytosis of chicken erythrocytes. At the molecular level, CFE promoted the expression of immunoglobulins (IgA, IgG, IgM) and cytokines (IL‐2, IFN‐γ). Our animal experiments thus demonstrated that CFE was safe and effective in improving immune function in mice. This suggests that CFE has potential for the treatment of immunocompromised patients, and as an adjuvant therapy for cancer patients receiving radiotherapy or chemotherapy and patients with other diseases that lead to immunosuppression, such as COVID‐19 and AIDS. However, the molecular biological mechanism by which CFE enhances immune function is not clear at present. Future studies should therefore examine the molecular mechanism of CFE.

## CONFLICT OF INTEREST

The authors declare no conflicts of interest.
