# Correction to “Condensed Fuzheng Extract Increases Immune Function in Mice With Cyclophosphamide‐Induced Immunosuppression”

**DOI:** 10.1002/fsn3.71905

**Published:** 2026-05-16

Wang, J. D., Wang, L., Yu, S., Jin, Y. T., Wang, Y. Y., Chai, R. D., Zhao, Z. Y., Bian, Y. H., & Zhao, S. W. (2022). Condensed Fuzheng Extract Increases Immune Function in Mice With Cyclophosphamide‐Induced Immunosuppression. Food Science & Nutrition, 10(11), 3865–3875. http://doi.org/10.1002/fsn3.2982

A concerned reader identified an error in the “RESULTS” section, subsection “3.2 Effect of CFE on Immunosuppressed Mice”, Figure 3. The authors re‐examined the published manuscript and the original experimental data and identified an error in Figure 3b: the representative thymus HE staining images for the CFE‐L and CFE‐M groups had been inadvertently misused. This mistake occurred during the process of assembling the figure and does not affect the results, analyses, or conclusions presented in the manuscript. The correct figure is provided below.

**FIGURE 3:**
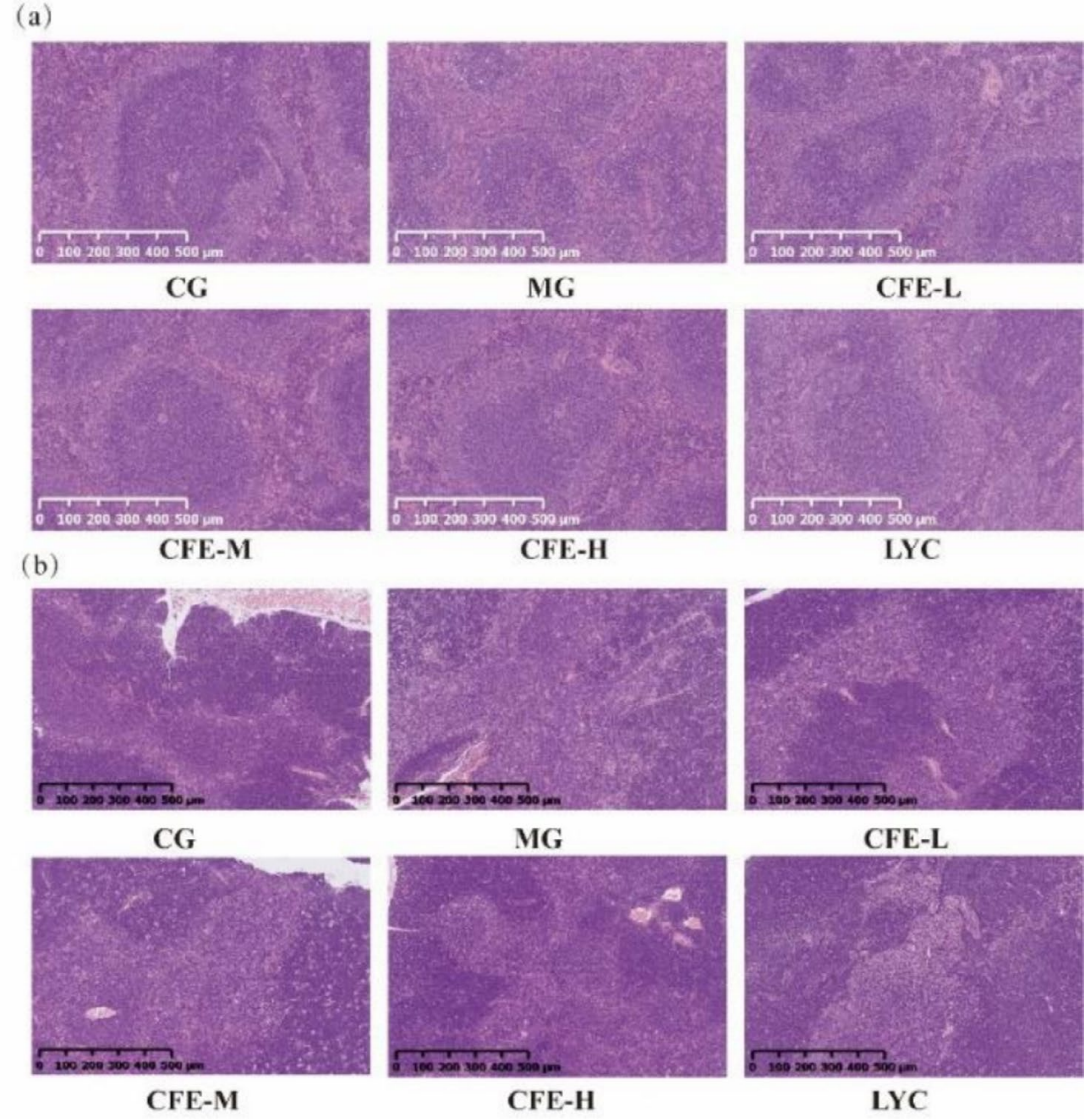
Effect of CFE on spleen histology (a) and thymus histology (b) of immunosuppressed mice.

We apologize for this error.

